# Investigating the need of triggering the acquisition for infant diffusion MRI: A quantitative study including bootstrap statistics

**DOI:** 10.1016/j.neuroimage.2012.11.063

**Published:** 2013-04-01

**Authors:** Lajos R. Kozák, Szabolcs Dávid, Gábor Rudas, Zoltán Vidnyánszky, Alexander Leemans, Zoltán Nagy

**Affiliations:** a MR Research Center, Semmelweis University, Budapest, Hungary; b Image Sciences Institute, University Medical Center Utrecht, Utrecht, The Netherlands; c Wellcome Trust Centre for Neuroimaging, UCL Institute of Neurology, London, UK

**Keywords:** ADC, apparent diffusion coefficient, bpm, beats/min, DTI, diffusion tensor imaging, DWI, diffusion-weighted imaging/diffusion weighted image, ECG, electrocardiogram, FA, fractional anisotropy, FOV, field of view, MRI, magnetic resonance imaging, NLLS, non-linear least squares fit, OLLS, ordinary linear least squares fit, (i)RESTORE, (informed) robust estimation of tensors by outlier rejection, SENSE, sensitivity encoding, SNR, signal-to-noise ratio, TR, repetition time, WLLS, weighted linear least squares fit, Diffusion weighted imaging, Diffusion tensor imaging, Magnetic resonance imaging, Artifacts, Image quality, Bootstrap statistics

## Abstract

Diffusion weighted magnetic resonance imaging is increasingly being used for neonatal and young pediatric subjects. Our purpose was to investigate a) whether cardiac triggering was needed to reduce variability of diffusion (tensor) imaging data, b) how pulsation artifacts affect the fitted diffusion tensor when triggering is not used and c) the feasibility of triggered data acquisition in neonates and young children.

Data were collected from 11 infants and 7 adults. In seven infants and seven adults, diffusion encoding was applied solely along the z gradient direction with and without cardiac triggering. Non-parametric bootstrap statistical methods were applied to investigate the dependence of variance on triggering. One infant and all adults served as test–retest controls. From the remaining three infants diffusion tensor imaging data were acquired with and without triggering.

Our findings that used the repeated measurements in a single diffusion-encoding direction indicated that without triggering the variability in the data was increased significantly both in infants and adults. When collecting diffusion tensor data in infants, this increased variability results in erroneous fractional anisotropy values and artifactual fiber direction estimates. Contrary to previous reports but supported by our findings involving adults, pulsation artifacts were present in a larger extent of the brain in the infant population.

In conclusion, triggering is feasible in young subjects and is preferred when acquiring diffusion MRI data. In doing so, the amount of erroneous estimations due to image artifacts will be minimized, which in turn will lead to more specific and less ambiguous interpretations. Although fitting the pulse-monitoring device requires additional set-up time, the total imaging time is usually shorter than acquiring multiple data sets to compile a single, artifact-free set.

## Highlights

► We investigated the need of triggering the diffusion MRI acquisitions in infants. ► Cardiac artifacts affect a larger portion of the infant brain than that of adults. ► In infants the time cost of cardiac triggering can be minimal compared to adults. ► The minimal overhead of ECG lead set up is outweighed by the improved data quality. ► Using cardiac triggering is feasible for improving diffusion imaging in infants.

## Introduction

**Fig. 1:**
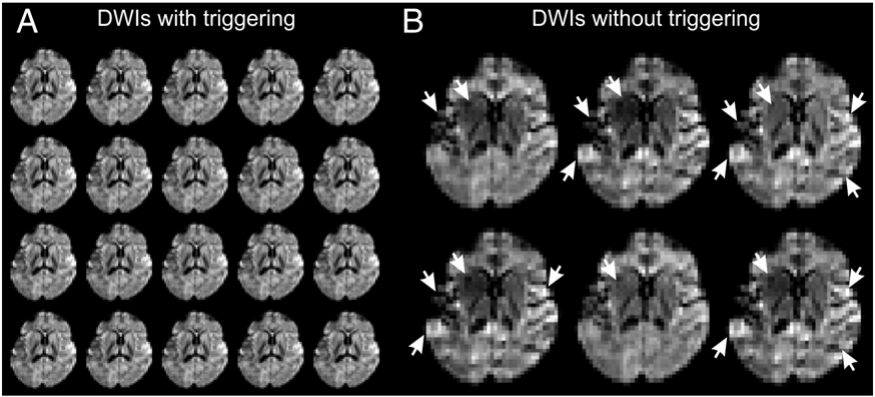
The effect of triggering. Representative DWIs from a single slice of one subject (4.8 months, male). For the triggered series all 20 images are shown, for the non-triggered series only images with the largest differences are shown as examples. Intensity scaling is arbitrary, but constant across images shown. (A) Demonstration of triggered acquisition resulting in low volume-to-volume variance with 20 consecutive images without artifacts. (B) Without triggering the volume-to-volume variance increases resulting in visible positive and negative differences in signal intensities. Six representative examples are shown with the most prominent artifacts marked with arrows (altogether 10 out of 20 consecutive images showed some artifacts).

Magnetic resonance imaging (MRI) is increasingly being used for neonatal and young pediatric subjects (Barkovich et al., 1988; Rutherford, 2002; Woodward et al., 2006). Often the examination protocol includes diffusion-weighted imaging (Le Bihan et al., 1986) to examine the white matter microstructure both in normal development (Lebel et al., 2008; Miller et al., 2003; Mukherjee et al., 2002; Neil et al., 1998) and in injured states (Huppi et al., 1998, 2001; Rutherford et al., 1991; van der Aa et al., 2011). However, diffusion weighted images (DWIs) are susceptible to several types of image artifacts which can be divided depending on whether the source is systematic or physiological (Tournier et al., 2011). Often, these artifacts are signal drop-outs (Fig. 1 and Video 1) and may occur due to cardiac pulsation (Wirestam et al., 1996). It has been shown in adults that pulse- or cardiac triggering (henceforth referred to as ‘triggering’ for brevity) improves the quality of diffusion-weighted data acquisition (Dietrich et al., 2000; Skare and Andersson, 2001).

Imaging neonatal subjects is extremely challenging because an awake baby will not stay still. Also, the heart rate of the newborns and young children can be consistently 120–160 beats/min (bpm) allowing little time for imaging between the heartbeats. Fitting sensors to detect heartbeats can also take precious examination time. For these reasons diffusion-weighted imaging of neonatal subjects usually proceeds without triggering.

For diffusion tensor imaging (DTI) the DWIs are usually collected along several non-collinear directions and used to estimate the apparent diffusion coefficient (ADC) (Le Bihan et al., 1986), diffusion anisotropy, e.g. fractional anisotropy (FA) (Basser and Pierpaoli, 1996), other measures of anisotropy (e.g., Frank, 2001), or carry out fiber tractography (Behrens et al., 2003; Conturo et al., 1999; Jeurissen et al., 2011; Jones et al., 1999; Mori et al., 1999). If motion induced signal reduction results in an overestimation of the ADC along one or more diffusion encoding directions, the 3-dimensional diffusion profile, along with the above-mentioned measures (e.g. FA), will be estimated inaccurately. In addition to the erroneous estimation of the diffusion properties in a given individual, these biases can propagate to group level comparisons (Chung et al., 2010; Zhu et al., 2009) if, by chance, the images of one group suffer more pulsation artifacts than the images of the other group.

The aims of this paper were to (a) examine the presence and extent of pulsation artifacts in DWIs collected from young pediatric subjects; (b) investigate the effect of pulsation artifacts on the estimated diffusion measures; and (c) test feasibility of triggered acquisition in this patient group.

## Patients and methods

### Subjects

Fifteen young children and infants were involved (age range 1–13 months, 10 girls, 1 preterm girl), each of which was in need of a clinical MRI examination. With the approval of the local ethics committee and after written informed consent of a parent, the clinical scanning session was supplemented with one of two acquisition protocols (described below). Additionally, twelve young adults (age: 23.3 ± 2.3 years, 3 females) were scanned with the approval of the local ethics committee and after written informed consent, to provide comparative adult data. All of the collected MRI data were first visually checked by at least one of two of the co-authors (L.R.K., Z.N.) for gross subject motion artifacts. Four infants (3 girls) and 5 adults (1 female) were excluded from further analysis due to excessive movement, thus we only report the results obtained on the remaining 11 infants and 7 adults (see Table 1 for details).

### Data acquisition

All images were collected on a 3 T Achieva Scanner (Philips Medical Systems, Best, The Netherlands) using an 8-channel receive-only head coil. According to local guidelines, the infants were sedated by qualified anesthesiologists using intra-venous propofol. The partial pressure of O_2_ and the heart rate of the subjects were constantly monitored during the sedation. The adult volunteers were scanned awake and without sedation. The vector electrocardiograph available on the Philips MRI scanners was fitted before the examination and used for monitoring the electrocardiogram (ECG) and for triggering.

Three experiments were performed using the Stejskal-Tanner (Stejskal and Tanner, 1965) diffusion encoding and echo-planar data collection methods. The common imaging parameters were: b-value = 0 s · mm^− 2^ for the reference image and either 1000 s · mm^− 2^ (2 infants and 7 adults) or 800 s · mm^− 2^ (9 infants) for the DWIs. If triggering was used, a single image was collected per heart-beat in infants while two images per heart-beat were collected in adults. The mean trigger delay with respect to the R-wave of the ECG (Skare and Andersson, 2001) was 202 ms (range: 202–250 ms) in infants, and 415 ms (range: 300–500 ms) in adults. Parallel imaging (SENSE (Pruessmann et al., 1999)) was employed with an acceleration factor of 2. The echo time was set to ‘shortest’ in the scanner software resulting in values in the range of 66–70.17 ms.

In *Experiment 1a*, two sets of 21 image volumes were collected from seven infants (4 females), one with and another without triggering. Each set contained one reference image and 20 DWIs with diffusion encoding along the z gradient (i.e. through-slice direction) axis only, in a fashion similar to Skare and Andersson (2001), because it maximizes sensitivity to the suspected movement of the brain due to the blood pulsation. Twenty-to-thirty slices were collected with a thickness of 3 mm and 1.5 mm gaps to provide full brain coverage. The field of view (FOV) was 240 mm with an in-plane resolution of 3 × 3 mm^2^. For one infant (1 month, female) a variant of *Experiment 1a* was performed, where *both* acquisitions were triggered to examine the test–retest variability.

In *Experiment 1b* three sets of 21 image volumes were collected from seven adults (2 females), the first and third with triggering while the second without triggering. Imaging parameters were otherwise the same as those of *Experiment 1a*.

In *Experiment 2*, two sets of 16 image volumes were collected from three infants (2 females), one with and one without triggering. Each set contained one reference image, followed by 15 DWIs with the diffusion directions distributed as implemented by Philips for diffusion tensor imaging. The slice thickness was 2.5 mm and 40 slices were collected, without gaps, to provide total brain coverage. The FOV was 200 mm with an in-plane resolution of 2.5 × 2.5 mm^2^.

In all experiments the total time of the first (triggered) acquisition was recorded and for the non-triggered acquisition the repetition time (TR) was set to give the same total acquisition time. Although usually triggering is avoided to minimize acquisition time, we controlled the TR to ensure that the extent of T1-relaxation was identical between the two datasets. If T1-relaxation is not controlled for, signal intensity, and in turn its variance estimate, can vary independent of the presence or absence of pulsation artifacts, confounding the results.

### Data processing

Image processing and statistical analyses were performed using in-house developed scripts in Matlab 7.5 (MathWorks Inc., Natick MA, USA), also utilizing the NIFTI image format manipulation routines of SPM (http://www.fil.ion.ucl.ac.uk/spm/).

**Fig. 2:**
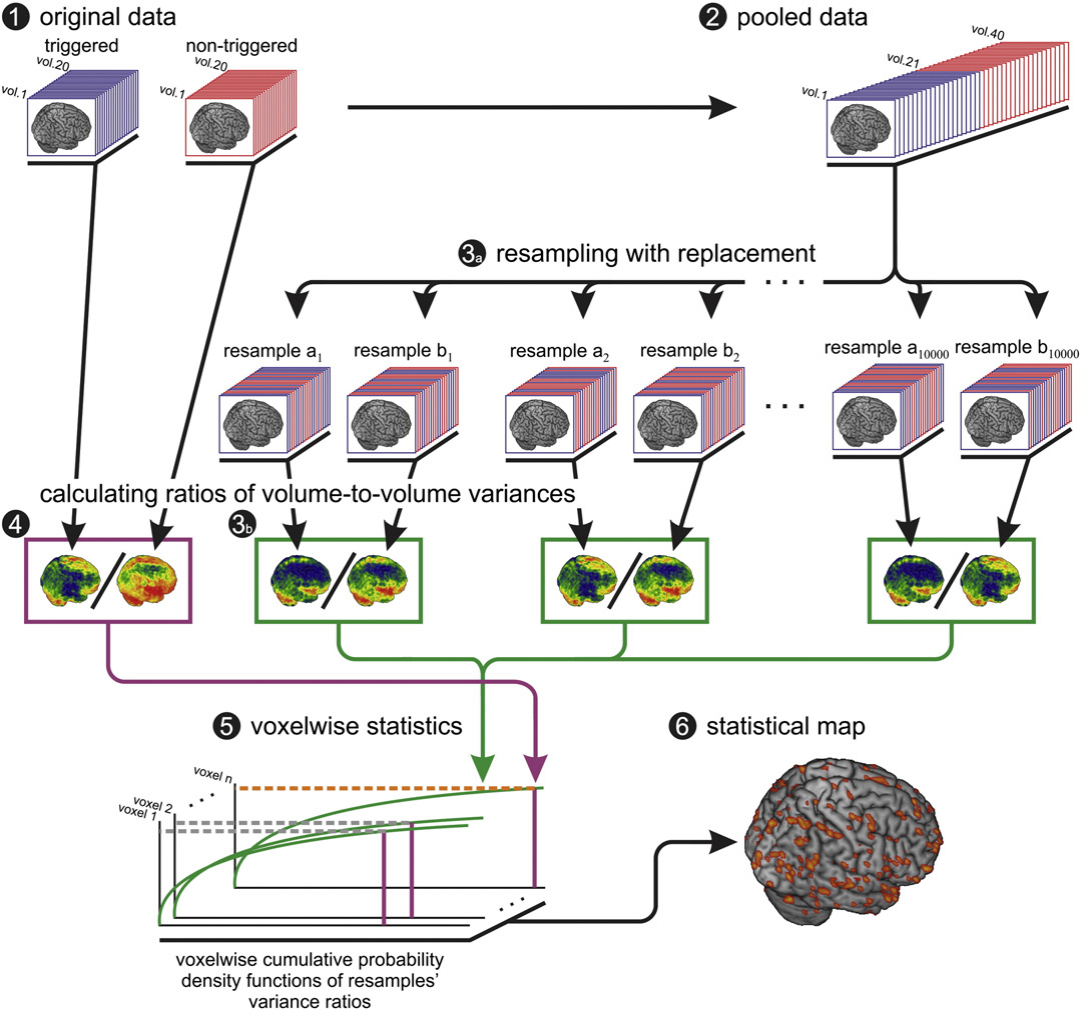
Flowchart of Bootstrap algorithm. Because the distribution of signal intensity in a given voxel over repeated measurements cannot be assumed to be normal the parametric F-test may not provide accurate p-values. This bootstrap procedure is the non-parametric equivalent of the parametric F-test and provides unbiased p values. Step 1) Obtain data with (blue, n^T^ = 20) and without triggering (red, n^N^ = 20). Step 2) Pool the triggered and non-triggerred data Step 3) randomly draw (with replacement) N = n^T^ + n^N^ = 40 images from the pooled data. Consider the first 20 images as a pseudo dataset with triggering, the other 20 as a pseudo dataset without triggering. Repeat this re-sampling procedure 10,000 times, calculate the variance for each of the resamples of pseudo triggered (*σ*_*b*1_^*T*^, *σ*_*b*2_^*T*^,…, *σ*_*b*10000_^*T*^) and pseudo non–triggered data (*σ*_*b*1_^*N*^, *σ*_*b*2_^*N*^,…, *σ*_*b*10000_^*N*^) and their respective ratios (*F*_1_^*b*^, *F*_2_^*b*^, … *F*_10000_^*b*^) and store the results. Step 4), calculate the variance of the original triggered (*σ*^*T*^) and the original non-triggered (*σ*^*N*^) dataset and their ratio F=σNσT. Step 5), compare the F value of the original data (F) to the distribution of 10,000 pseudo *F*^b^ values obtained from the bootstrap re-sampling procedure. Step 6), identify voxels where fewer than 5 of the 10,000 pseudo *F*^b^ values are larger than the original F value. This corresponds to p < 0.0005 meaning that if the triggered and non-triggered data were drawn from the same distribution, there is less than 5 in 10,000 chance to obtain an F value that is as large; i.e. in a given voxel where there is no difference in variance there is only a 5/10000 chance that we would say there is.

The data from *Experiments 1a&b*, were put through a bootstrap statistical procedure, see below and in Fig. 2, for testing the effect of triggering on data variance.

To the data collected in *Experiment 2* the diffusion tensor was fit (Basser and Pierpaoli, 1996) using ordinary linear least squares (OLLS), weighted linear least squares (WLLS), and non-linear least squares (NLLS) models as implemented in the ExploreDTI software (http://www.exploredti.com/) to test the effect of triggering on diffusion tensor parameters. From the tensor, FA images were calculated and the x, y, and z components of the eigenvector corresponding to the largest eigenvalue (Anton, 2005) were color coded to represent fiber directionality (Pajevic and Pierpaoli, 1999). The informed RESTORE (iRESTORE) algorithm (Chang et al., 2012) was also applied to the non-triggered data sets to evaluate its effectiveness in eliminating the effects of the artifacts on the tensor fit as compared to pulse triggering. Before diffusion tensor fitting, all DTI datasets were corrected for motion and eddy current induced distortions (Leemans and Jones, 2009).

### Statistical methods

A bootstrap statistical procedure (Efron and Tibishirani, 1998) was performed on the data from *Experiments 1a&b* to examine whether the variance was systematically reduced in the triggered data (Nagy et al., 2008). Usually, for evaluating the difference of variances between two groups the F-test is employed (Rosner, 2000), but this parametric statistical test is sensitive to deviations from the normal distribution. While, given enough data, the central limit theorem (Freund et al., 1999) assures that the sampling distribution of the mean is normal regardless the population's distribution, no such theorem exists for variances. Indeed, as shown by simulations, the shape of the distributions have dramatic effects on the precision of the F-test (Ott and Longnecker, 2010), thus sampling a population that is not normally distributed can result in unreliable p-values.

As the noise in MR images is not normally distributed (Haacke, 1999), especially in the low signal-to-noise domain, which is typically the case with DWIs, the theoretical F-distribution is not appropriate for testing variances. This is the reason for using the bootstrap procedure (Efron and Tibishirani, 1998), in this case set up as a non-parametric variant of the F-test where the population distribution is simulated from the available data by re-sampling it with replacement (for implementation details see Fig. 2.)

Effect sizes were calculated by extracting voxel-wise variances either from the whole brain or only from those voxels of the original non-resampled data that were deemed to be significantly different by the bootstrap. The ratio of variances of the non-triggered and triggered datasets was calculated voxel-wise and then concatenated across voxels and subjects to form an empirical distribution of variance ratios (i.e. F-values). As this distribution is highly skewed the mean is not representative, instead the median and the interquartile range will be given.

## Results

### *Experiments 1a&b* — Investigating the effect of triggering on the variance of the data

**Fig. 3:**
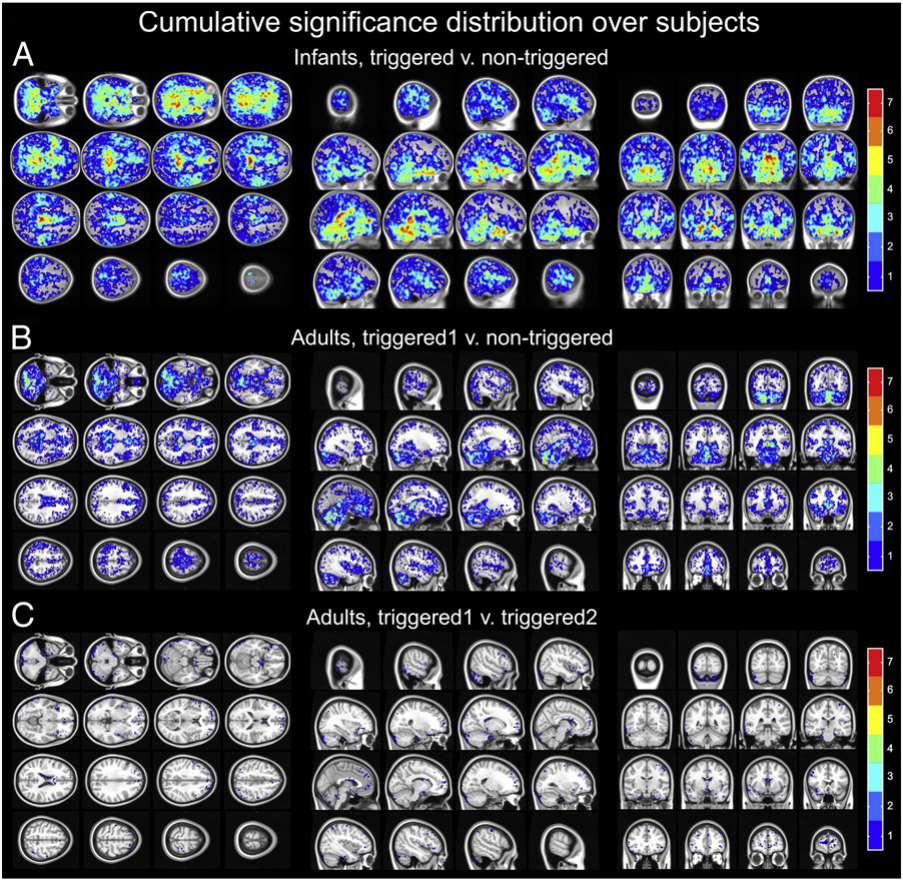
Significant differences between triggered and non-triggered acquisitions. Cumulative distribution of significances was calculated by summing the thresholded (p < 0.0005, uncorrected) co-registered significance maps of the subjects. Any given color-coded voxel was statistically significant in at least 1 subject (blue) but possibly in all subjects (red). The infant map is shown projected onto the Cincinnati Children's Hospital Medical Center infant brain template (https://irc.cchmc.org/software/infant.php), the adult map is projected to the non-linear 1 mm MNI152 template of the FMRIB Software Library (http://fsl.fmrib.ox.ac.uk/fsl/) (A) Significant differences between triggered and non-triggered acquisitions in infants. (B) Significant differences between the first triggered and the non-triggered acquisition in adults. Comparing the non-triggered acquisition to the second triggered acquisition lead to similar results (not shown but see Table 2 for quantitative description) (C) Results of the control experiment where the two triggered acquisitions were statistically compared in adults.

The variance in the acquired data is higher when triggering is not used regardless of the investigated population. Often this can be easily identified upon visual inspection (see Fig. 1 and Video 1), nevertheless bootstrap statistics help establishing the significance of differences. Indeed, bootstrap results from seven infants show large areas where the voxel-wise variance in the repeated measurement of diffusion along the z gradient axis is significantly reduced (p < 0.0005) when triggering is used (Fig. 3A). The minimum effect size over significant voxels was 2.1, meaning the non-triggered data had at least twice the variance; the median effect size was 19.3, for more details see Table 2.

Note that, the distribution of loci where increased variance is found in the non-triggered data is extensive in this group of young subjects with significant differences present bilaterally not confined to CSF spaces, but also affecting both grey and white matter, even in the superior slices. This is in contrast both with previous observations in adults (Skare and Andersson, 2001), and our results from *Experiment 1b*, where pulsatile artifacts tend to be confined to CSF spaces and to central basal brain areas (Fig. 3B). Moreover, the median effect sizes over significant voxels were only half as large in adults (10.5 and 9.9 for the first and second triggered acquisition compared against the non-triggered, respectively), for detailed effect size calculations, see Table 2.

To internally validate the methods the same bootstrap statistics was applied to repeated measurements with triggering in a single infant, and in all adults. This resulted in a much smaller number of and more scattered voxels both in the infant and in the adults that survived the same threshold of statistical significance (infant data not shown, for adult group data see Fig. 3C).

### *Experiment 2* — Investigating the effect of triggering on the diffusion tensor

**Fig. 4:**
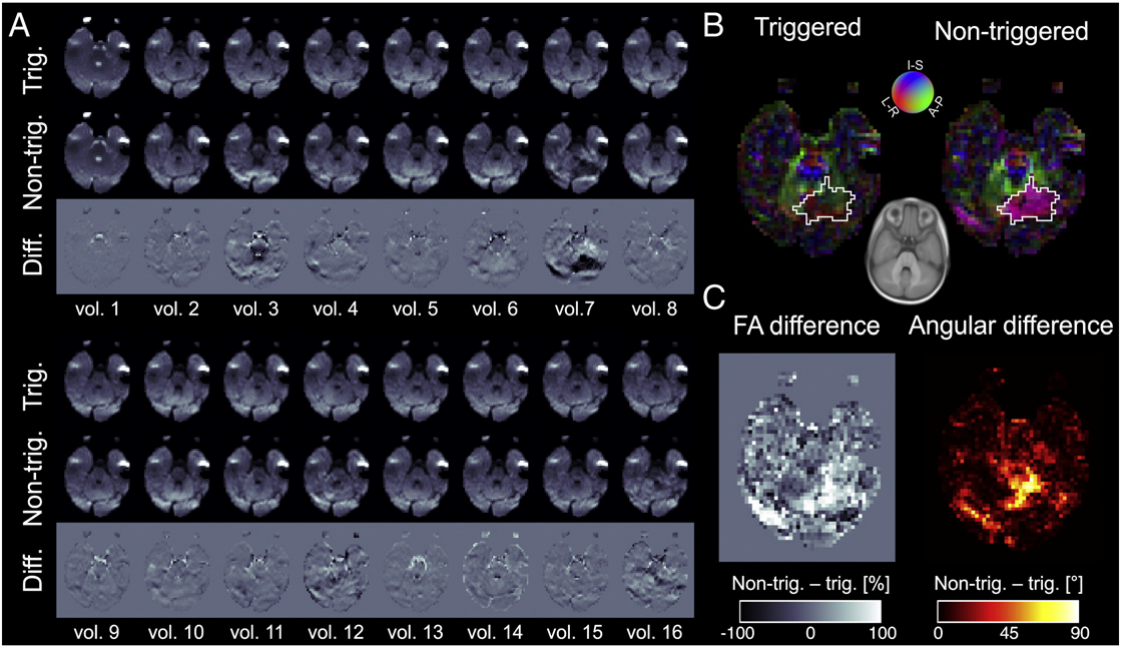
Pulsation artifacts affect the tensor estimation. (A) Volume-wise comparison of DWIs shows clear differences between triggered and non-triggered acquisitions. (B) The differences are propagated to the calculated (OLLS) tensor parameters, as seen on the FA modulated colored orientation maps. A coregistered slice of the Cincinnati Children's Hospital Medical Center infant brain template (https://irc.cchmc.org/software/infant.php) is shown in inset as reference for anatomical localization. (B) FA difference maps, and the principal eigenvectors' angular difference maps highlight areas most affected by pulsation artifacts. Representative data from a single subject, the angular difference maps are modulated by the FA difference map. See text for quantitative analysis of the data in the region of interest outlined by white.

Cardiac pulsation affects both the acquired DTI data, and the tensor fitting results. For the qualitative effects of pulsatile artifacts see Fig. 4A which displays all the acquired DTI data, both with and without triggering, in a single representative slice from one subject (female, age: 10.1 months, heart rate: 132 bpm). There is visible variability even in the triggered data, however this is expected as the diffusion-encoding directions change from volume-to-volume and thermal noise is also present. When the corresponding images with identical diffusion-encoding directions are subtracted large, connected areas become visible which have non-anatomical arrangements — that is they are likely to be due to pulsatile artifacts.

If the tensor model is fit to the above two datasets both the FA values and the fiber orientations can attain artifactual values that sometimes are clearly visible (Figs. 4B & C), but sometimes can be very hard to identify upon visual inspection as erroneous. E.g., the large artifact seen on DWI vol. 7 leads upon OLLS tensor fitting to the large purple-colored region of interest (marked with a white outline in Fig. 4B), where FA values are significantly different between the non-triggered (0.69 ± 0.21) and triggered (0.37 ± 0.11) acquisitions (p < 0.0001, paired Student's t-test).

**Supplementary Fig. 1:**
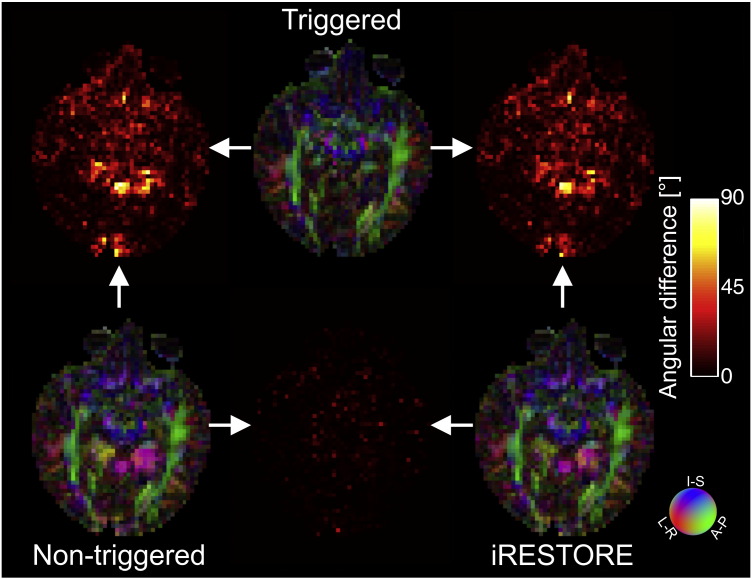
Color coded maps of the orientation of the principal eigenvector are displayed for the triggered (top middle) and non-triggered acquisitions obtained using OLLS tensor fitting (bottom left), as well as for the non-triggered acquisition after employing the iRESTORE algorithm (bottom right). Paired angular difference images of the principal eigenvectors' orientations are also presented. The white arrows indicate which of the acquisition/processing methods were compared; the angular difference maps are modulated by the respective FA difference maps.

**Supplementary Fig. 2:**
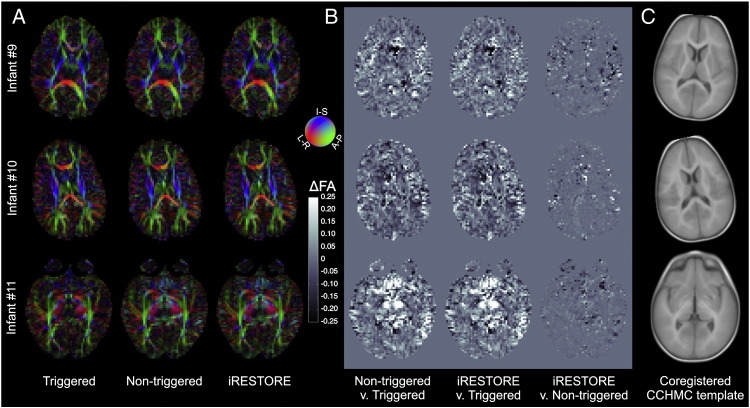
Representative slices from 3 subjects with visible artifacts on the colored orientation maps. (A) Color coded maps of the orientation of the principal eigenvector are displayed for the triggered (left column) and non-triggered acquisitions obtained using OLLS tensor fitting (middle column), as well as for the non-triggered acquisition after employing the iRESTORE algorithm (right column). (B) Fractional anisotropy difference images for non-triggered v. triggered (left column), non-triggered with iRESTORE-applied v. triggered (middle column), and non-triggered with v. without iRESTORE (right column).The iRESTORE algorithm corrects some of the artifacts that are present in the non-triggered data, nevertheless some artifacts remain uncorrected (see the rightmost column) in all examinations. (C) Coregistered slices of the Cincinnati Children's Hospital Medical Center infant brain template (https://irc.cchmc.org/software/infant.php) are shown as references for anatomical localization.

**Fig. 5:**
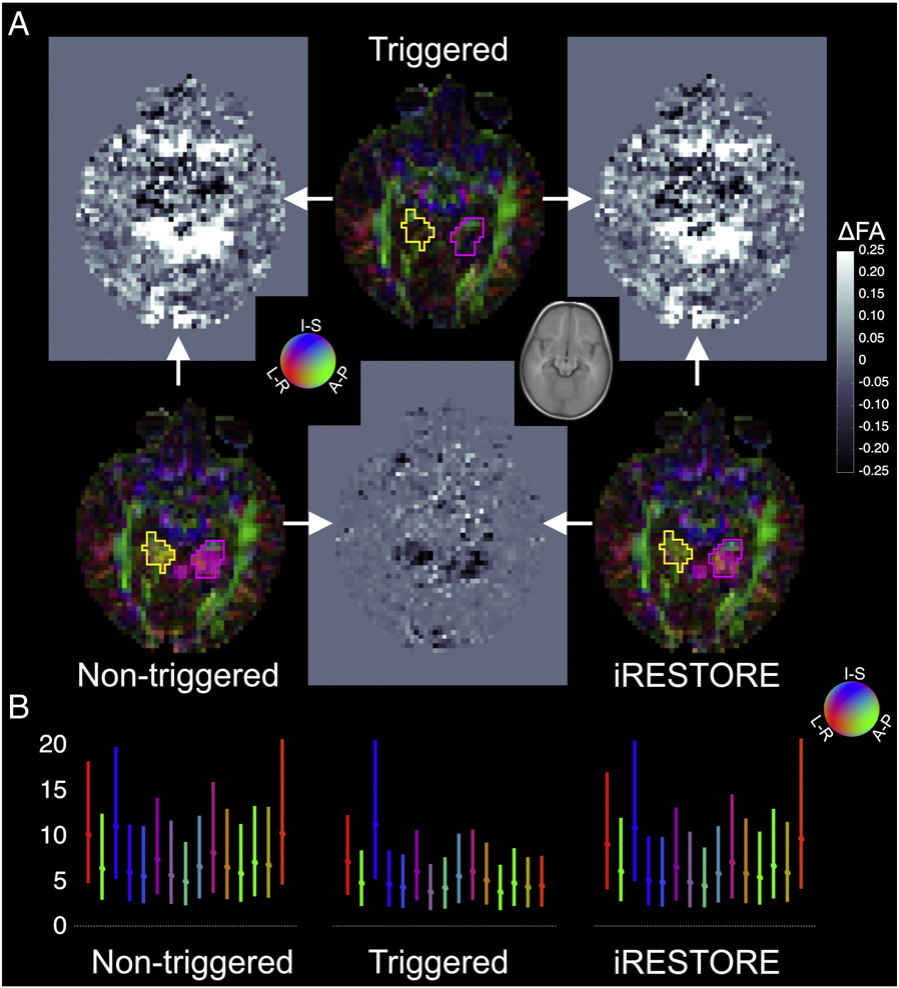
The iRESTORE algorithm provides partial remedy for pulsation artifacts. (A) Color coded maps of the orientation of the principal eigenvector are displayed for the triggered (top middle) and non-triggered acquisitions obtained using OLLS tensor fitting (bottom left), as well as for the non-triggered acquisition after employing the iRESTORE algorithm (bottom right). The iRESTORE algorithm corrects some of the artifacts that are clearly present in the non-triggered data set without employing the algorithm. Still the triggered data set provides the most reliable results, Paired fractional anisotropy difference images are also presented among the 3 methods. The bottom middle figure depicts the improvement due to the iRESTORE algorithm. The white arrows indicate the acquisition methods compared. A coregistered slice of the Cincinnati Children's Hospital Medical Center infant brain template (https://irc.cchmc.org/software/infant.php) is shown in inset as reference for anatomical localization. For comparison of the principal eigenvectors' orientation, see Supplementary Fig. 1. (B) Mean (and range) of absolute residuals of OLLS tensor fit errors for the non-triggered, triggered and non-triggered with iRESTORE experiments. The y axis scale is arbitrary but identical for all 3 plots while the x axis represents the 15 diffusion encoding directions. Note that mean residual is largest for the 3rd diffusion direction, which is along z gradient axis. The bars are color coded according to the diffusion encoding direction using the standard DTI color scheme (see inset). See Results for quantitative analysis of the error introduced in FA values and the principal tensor directions within the regions of interest outlined on the color coded maps.

Non-triggered acquisitions have larger mean absolute residual errors in the diffusion tensor fits than triggered acquisitions regardless of the fitting method used (Table 3). Applying the iRESTORE approach (Chang et al., 2012) to the non-triggered data improves the results by decreasing these errors, and by partially correcting the excessive FA values and the incorrect tensor directionality (Fig. 5 and Supplementary Figs. 1 and 2). Despite these improvements the absolute residual errors are bigger and more extensive, and the FA values and tensor directionality are still artifactual in larger areas of brain tissue for non-triggered data than what can be achieved by the triggered acquisitions. E.g., the FA values in the region interest outlined by a thin yellow line in Fig. 5 are 0.35 ± 0.16 for the triggered acquisition (OLLS fit), 0.70 ± 0.15 for the non-triggered acquisition with OLLS fit, and 0.59 ± 0.10 for the non-triggered acquisition with iRESTORE fit (p < 0.0001, one-way ANOVA; all groups are significantly different on pairwise post hoc comparisons at p < 0.05, with Tukey HSD test). The mean orientation of the principal eigenvectors differ by 27.0° in the triggered v. non-triggered (OLLS fit) and 28.5° in the triggered v. non-triggered (iRESTORE fit) comparison; the difference between the two non-triggered fits is 4.3°. Similar effect of pulsation is visible in the region of interest outlined by a thin purple line in Fig. 5 where FA values are 0.38 ± 0.16 for the triggered acquisition (OLLS fit), 0.75 ± 0.16 for the non-triggered acquisition with OLLS fit, and 0.65 ± 0.14 for the non-triggered acquisition with iRESTORE fit (p < 0.0001, one-way ANOVA; the triggered data is significantly different from the non-triggered ones on pairwise post hoc comparisons at p < 0.05, with Tukey HSD test). In this ROI the mean orientation of the principal eigenvectors differ by 84.5° in the triggered v. non-triggered (OLLS fit) and 92.3°in the triggered v. non-triggered (iRESTORE fit) comparisons; the difference between the two non-triggered fits is 8.2°.

Note that the mean residuals for the OLLS diffusion tensor fit are largest when diffusion encoding is along the z gradient axis (3rd bar from the left in blue in each plot of Fig. 5B). This result supports using the z gradient axis for experiment 1 but there is a tendency for a higher mean residual also along the x gradient axis (1st bar from left in each plot of Fig. 5B).

## Discussion

Taken together, our results indicate that imaging neonatal subjects without triggering the acquisition results in increased variance in the data, causing a severe bias in the estimated diffusion (tensor) parameters in a larger portion of the brain than that in adults.

The identification of pulsatile artifacts in diffusion-weighted brain image data is not new (Wirestam et al., 1996). It has been demonstrated previously in adults and triggering has already been suggested as a remedy (Dietrich et al., 2000; Gui et al., 2008; Nunes et al., 2005; Skare and Andersson, 2001). However, the acquisition of diffusion data in neonates usually proceeds without triggering and a systematic investigation whether triggering is beneficial in this patient group has never been performed to date. Here, we investigated pulsatile artifacts and demonstrated that these artifacts are more widespread in infants than it would be expected from adult data (Skare and Andersson, 2001). In addition, we showed that triggering is a remedy for these widespread artifacts, as it significantly decreases their presence. The cause of the differences in the distribution of pulsatile artifacts between infant and adult brains is still unclear. It may be due to a) the infants' higher heart rate; b) the fact that the vasculature is not yet fully developed and may be more compliant; c) the larger water percentage of infant brain tissue; d) the larger relative size of the skull compared to the size of the brain in infants; and e) the more flexible skull of infants.

**Supplementary Fig. 3:**
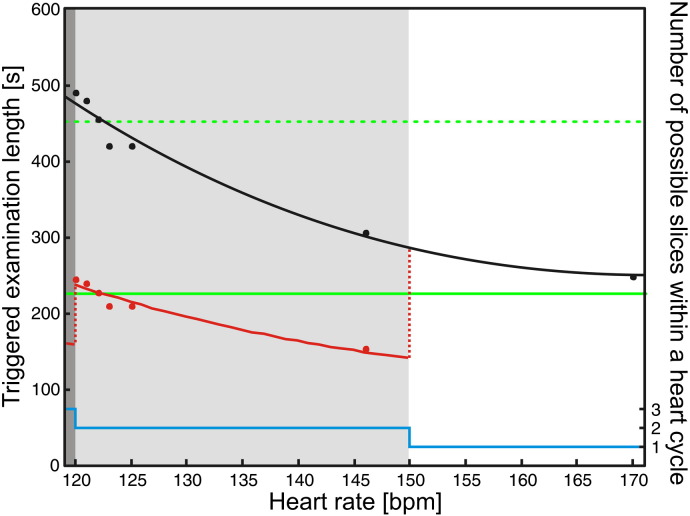
Triggering increases examination time in a heart-rate dependent fashion. Slower heart rates result in higher examination times when acquisitions are triggered. The length of non-triggered acquisition with TR = 8000 ms (3:46) is shown as solid green line; the total time for two back-to-back acquisitions is shown as dotted green line. With the single slice per heart cycle strategy we used (black dots), examination length increases to more than double of that of the non-triggered acquisition if average heart rate falls below 123 beats/min (bpm) as visible from a second order polynomial fit shown as solid black line. However as the acquisition time of a single slice is about 100 ms on our 3 T scanner, 2 slices could be collected in a single heart cycle if the heart rate is between 120 and 150 bpm, and 3 slices if the heart rate is below 120 bpm. The total expected examination time with multiple acquisitions in a single heart cycle is depicted as red dots, and a second order polynomial fit represented by a red line. Note, that these expected values are valid for a trigger delay of 200 ms.

One argument against triggering could be that the extended acquisition time allows for more subject movement, introducing further variance in the data. This argument however is not necessarily valid for young pediatric patients, where the examinations are usually performed while asleep or under sedation. Moreover, this limitation is only true if the strategy is to collect single images per heart cycle. On our 3 T system, a single DWI is collected in 100 ms, so theoretically more slices could be collected in a heartbeat (see Supplementary Fig. 3), similar to the approach of Chung et al., 2010. However, further investigation is needed to determine the optimal time window for low-artifact image collection.

Another argument against triggering could be the time penalty of setting up the ECG leads. However, this can be achieved in less than a minute in parallel with other aspects of patient preparation (induction of sedation, setting up other monitoring devices, e.g. pulse oximeter, etc.).

As the brains of neonates and young children are relatively small, a volume-to-volume minimum TR of only 2–3 s is sufficient to achieve full-brain coverage. Because such a short TR does not allow appropriate relaxation of the longitudinal magnetization, investigators usually do not collect data with this setting, instead the TR is set to about 6–8 s (Anjari et al., 2007; Counsell et al., 2006; Deipolyi et al., 2005; Hermoye et al., 2006). Nevertheless, one could acquire all of the images twice with the minimal TR of e.g. 3 s and average them for a $\sqrt{2}$ gain in signal-to-noise ratio (SNR), without increasing the acquisition time. Assuming a T1 relaxation time of 1700 ms for white matter (Jones et al., 2004), the amount of relaxation and hence the available signal strength would then increase from about 83% to 97%. At first sight, this might seem to be an advantageous approach, however, if artifacts occur, averaging could not be done and the gain in SNR would not be achieved, leading to a dataset of varying SNR. The same issue with directionally varying SNR is true for the practice of collecting non-triggered datasets multiple times with 6-8 s TR and then subsequently compiling a single set of artifact-free images (Mori et al., 2002; Simonyan et al., 2008). Moreover, the latter approach doubles acquisition time.

Another strategy is to use outlier rejection methods (Chang et al., 2005, 2012; Morris et al., 2011), where in each voxel, the diffusion tensor is fit to only those diffusion-encoding directions that fit the model well. Performing informed RESTORE (Chang et al., 2012) on our data improves the results significantly but residual artifacts remain (see Fig. 5 and Supplementary Figs. 1 & 2). This is most likely due to the limited amount of high quality diffusion weighted volumes. Using 12–15 diffusion direction is common practice in the pediatric population (Bassi et al., 2011; Bednarek et al., 2012; Skiold et al., 2010), and as our results suggests the tensor estimates can be biased even after the exclusion of outliers (see also the Discussion in Chang et al., 2005, 2012). Note, however, that there are studies with neonatal participants that proceed with the minimally needed 6 diffusion directions (Seghier et al., 2005). In this case, outlier rejection methods are not possible because the exclusion of a single direction removes the possibility of fitting a tensor to the remaining data (note that there are no non-zero residuals in the first place). Only a few research groups have now started to collect data with up to 42 directions (Dudink et al., 2011; van der Aa et al., 2011; van Kooij et al., 2012), in which case outlier rejection methods may be a viable alternative to pulse triggering for diffusion tensor imaging. If the larger number of diffusion-encoding directions are used to fit a more complex model, the redundancy of the data will be reduced (i.e., larger number of parameters need to be estimated). As pulse triggering carries less of an overhead in acquisition time for infants than in adults, and given the fact that most vendors do not support robust diffusion tensor estimation procedures, such as (i)RESTORE, triggering is especially recommended in a clinical setting.

Triggering has the composite benefit that it helps eliminating artifacts from the data while increasing the volume-to-volume TR, thus allowing for more complete T1-relaxation and optimum SNR in an artifact free data set. For 20 slices and using the parameter set of this study, the minimum TR was 2.1 s whereas the effective TR for the triggered acquisition was approximately 10.0 s allowing for 71% and ~ 100% T1-relaxation, respectively. Note that this fortunate scenario only occurs in this particular target group. In older subjects with larger brains, shorter T1-relaxation, and slower heart rate, the minimum volume-to-volume TR may allow for complete T1-relaxation; therefore triggering per se does not necessarily result in an increased SNR.

It must also be noted that the sensitivity of different reconstruction schemes to pulsatile motion of the brain is variable (Robson and Porter, 2005). If in doubt, and if the population is available, the bootstrap procedure can be easily implemented to ascertain the local need for triggering. However, as triggered acquisitions in infants hardly increase examination time, we would recommend using triggering in general, unless evidence is available to the contrary.

The main limitations of our study stem from the extremely limited access to our target infant pediatric population; this is the reason why (a) the bootstrap procedure was only applied to repeated measurements with diffusion-encoding along the z gradient direction but not on diffusion tensor data; (b) repeated measurement with triggering was not performed for the diffusion tensor imaging. One can argue that our decision of collecting data for the bootstrap statistics with 1.5 mm gaps in order to reduce examination time is another limitation, but as the observed pulsation artifacts have larger extent than a single slice thickness the gaps did not limit the sensitivity of the method, i.e., it is not likely that false negatives could have occurred due to missing data in the gaps.

## Conclusion

Cardiac triggering is a feasible approach for improving the quality of diffusion-weighted MR images in infants as it effectively decreases circulation-related artifacts with a negligible increase in acquisition time.

The following are the supplementary date related to this article.

Video 1 Differences between triggered and non-triggered data sets in Experiment 1. Representative DWIs with diffusion-encoding along the z gradient axis from the same subject as in Fig. 4 (4.8 .months, male). The left and right panels show the non-triggered and triggered data sets respectively. Intensity scaling and windowing are arbitrary, but constant across images shown.

## Figures and Tables

**Table 1:** Description of infants and adults involved.

		Age (months)	Sex	Start HR (bpm)	b-value	Reason for exam	Actual finding
Bootstrap (infant)	#1	4.8	M	146	1000	Orbital tumor	Normal
#2	5.1	F	170	800	Epilepsy	Normal
#3	7.8	F	121	800	Obstructive hydrocephalus	No obstruction
#4	6.0	F	122	800	Ewing sarcoma	Progression
#5	9.6	M	125	800	Oseomyelitis	Improvement
#6	8.6	F	123	800	Hemiparesis	Left periventricular cyst, white matter loss
#7	8.6	M	120	800	Orbital tumor	Bone metastasis of neuroblastoma
#8	1.1	F	160	1000	Congenital malformation	Stroke
DTI	#9	10.1	F	132	800	Neuroblastoma follow-up	No progression
#10	9.3	M	135	800	Neuroblastoma follow-up	Normal
#11	13.3	F	137	800	Synovial sarcoma follow-up	Normal


		Age (years)	Sex	Start HR (bpm)	b-value	Reason for exam	Actual finding

Bootstrap (adult)	#1	21.3	M	85	1000	N/A	N/A
#2	21.9	M	70	1000	N/A	N/A
#3	22.4	F	70	1000	N/A	N/A
#4	21.4	M	80	1000	N/A	N/A
#5	26.9	M	70	1000	N/A	N/A
#6	27.9	F	60	1000	N/A	N/A
#7	24.9	M	70	1000	N/A	N/A

**Table 2:** Mean effect sizes as assessed by variance ratio calculation over subjects.

	Over whole brain		Over significant voxels	
	Median	Interquartile range	Median	Interquartile range
Infant triggered v. non-triggered	3.6	1.5–11.4	19.3	9.3–49.4
Adult triggered1 v. non-triggered	2.2	1.0–5.4	10.5	5.9–23.0
Adult triggered2 v. non-triggered	2.6	1.2–6.3	9.9	5.6–21.9
Adult triggered1 v. triggered2	1.2	0.7–1.9	0.2	0.1–0.3

**Table 3:** Mean absolute residuals of model errors upon diffusion tensor fitting. OLLS: ordinary linear least squares fit; WLLS: weighted linear least squares fit; NLLS: non-linear least squares fit; iRESTORE: informed RESTORE.

		Infant #09		Infant #10		Infant #11	
		99% range	Median	99% range	Median	99% range	Median
Triggered	OLLS	2.1–30.6	5.95	2.4–33.3	6.86	1.6–28.6	4.99z
WLLS	2.1–30.2	5.90	2.4–34.1	6.80	1.6–28.2	4.95
NLLS	2.1–30.0	5.91	2.4–32.1	6.82	1.6–27.6	4.96
Non-triggered	OLLS	2.6–63.3	8.31	2.8–43.6	8.84	2.0–33.0	6.16
WLLS	2.6–57.0	8.23	2.9–42.4	8.83	2.0–31.5	6.12
NLLS	2.6–57.8	8.27	2.9–41.4	8.81	2.0–31.5	6.13
iRESTORE	2.5–56.5	8.00	2.8–40.1	8.56	1.9–30.2	5.95
